# Deformation and Failure Behavior of Wooden Sandwich Composites with Taiji Honeycomb Core under a Three-Point Bending Test

**DOI:** 10.3390/ma11112325

**Published:** 2018-11-19

**Authors:** Jingxin Hao, Xinfeng Wu, Gloria Oporto, Jingxin Wang, Gregory Dahle, Nan Nan

**Affiliations:** 1 College of Material Science and Engineering, Central South University of Forestry and Technology, Changsha 410004, China; haojingxin1@163.com (J.H.); wuxinfeng1@126.com (X.W.); 2 Division of Forestry and Natural Resources, West Virginia University, Morgantown, WV 26506, USA; gregory.dahle@mail.wvu.edu (G.D.); nnan@mix.wvu.edu (N.N.)

**Keywords:** honeycomb, composite, wood-based materials, failure map, core shear failure, indentation

## Abstract

A new type of Taiji honeycomb structure bonded outside with wood-based laminates was characterized from a mechanical standpoint. Both theoretical and experimental methods were employed to analyze comprehensively the deformation behavior and failure mechanism under a three-point bending test. The analytical analysis reveals that a Taiji honeycomb has 3.5 times higher strength in compression and 3.44 times higher strength in shear compared with a traditional hexagonal honeycomb. Considering the strength-weight issue, the novel structure also displays an increase in compression strength of 1.75 times and shear strength of 1.72 times. Under a three-point bending test, indentation and core shear failure played the dominant role for the total failure of a wooden sandwich with Taiji honeycomb core. Typical face yield was not observed due to limited thickness-span ratio of specimens. Large spans weaken the loading level due to the contribution of global bending stress in the compressive skin to indentation failure. A set of analytical equations between mechanical properties and key structure parameters were developed to accurately predict the threshold stresses corresponding to the onset of those deformation events, which offer critical new knowledge for the rational structure design of wooden sandwich composites.

## 1. Introduction

The increasing need for cost-effective construction materials, together with the decreasing availability of raw materials, triggers the research, development and innovation in the composites field direction. Because of their high stiffness-to-weight and strength-to-weight ratios, sandwich composites have been receiving important attention in the past few years [1,2]. In general, a sandwich composite is composed of two external faces or flanges and one internal layer or core.

The diverse raw materials used in the face and core layers of a sandwich composite make their mechanical properties, such as strength and stress deformation, more complex than homogeneous materials [3,4]. Previous theoretical and experimental studies have examined failure behavior of composite sandwich beams under static flexural tests [5,6]. Reference [7] for instance, found face yield in sandwich beams with carbon/epoxy facings and aluminum honeycomb core loaded in four-point bending. As for sandwich composites subjected to a three-point bending test, the dominant failure modes were core shear failure and surface indentation [8,9,10]. Reference [11] generated collapse mechanism maps for sandwich composites in bending to show the dependence of the failure mode upon the geometry of the beam and the relative strength of the faces and core. These composites included aluminum alloy face-sheets and polymeric foam-cores, or metallic face-sheets and metallic foam-cores. To make an accurate prediction of static failure loads and modes, others attempted to consider a local deflection effect near loading point [12,13,14,15,16]. Because of the complexity of solutions, a limited failure mode map has been constructed. In some special cases, face sheet debonding may be developed because of embedded defects during the fabrication of sandwich panels. Debonding reduces the stiffness and strength of the sandwich structure so that it will be fractured in the relatively low loading level. However, this type of failure has not typically been observed in many sandwich beam specimens under usual quasi-static loading configuration [17,18].

A wooden sandwich composite with a paper honeycomb core has the potential to replace partially conventional thick and heavy solid boards including medium density fiber board, particle board, and plywood used in the furniture, interior decoration and packaging fields etc. [19,20]. However, there are still some limitations on the use of a paper honeycomb core in sandwich composites, mostly related to their load bearing resistance [19,21]. Reference [22]. developed a hexagonal honeycomb fortified by wooden strips with improved compression strength; however, due to the composite complexity and high production costs, the composite was not commercially successful. Developing a new honeycomb construction with high strength is critical to improve the total mechanical properties of this kind of sandwich composite.

The main goal of this research was to investigate mechanical properties of a new light-weight sandwich composite constituted by a Taiji honeycomb core between two layers of woody based-composites (medium density fiberboard and plywood). The deformation and failure mechanism of the new type of composite was investigated under a three-point bending test by employing both theoretical and experiment methods. To identify as many possible failure modes, specimen’s combination of various faces and core thickness, in addition to the span distances, were evaluated. A general static failure mechanism map was delineated using the transition equations between the failure modes. The analytical comparison between the Taiji honeycomb and a traditional hexagonal core was also presented.

## 2. Experiments

### 2.1. Composite Design

Sandwich composites prepared for this research consisted of two wooden face sheets glued with a paper honeycomb core. The following three types of surface sheets were used: 3.175 mm thickness of medium density fiber board (MDF, Masonite International Corporation, Tampa, FL, USA), 3.175 mm thickness of plywood (PLY, Woodcraft, Parkersburg, WV, USA) and 6.35 mm thickness of plywood (Woodcraft Supply, Parkersburg, WV, USA). All of them have been used effectively for wooden-panel furniture and package products. Preliminary tests to characterize these materials in terms of their physical and mechanical properties were conducted using ASTM D1037-06a [23]. The results are presented in Table 1.

**Figure 1:**
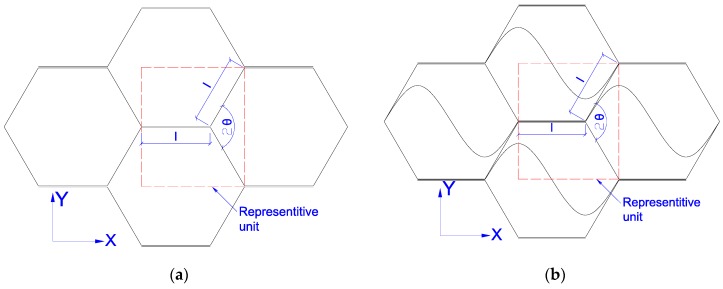
The idealized structure of honeycomb core: (**a**) Traditional hexagonal honeycomb, (**b**) Taiji honeycomb.

To date, the commonly used honeycomb structures, such as paper honeycomb and aluminum honeycomb, are the ones whose cell unit comprises a double layer of ribbon edge and two single layers of inclined edge, as presented in Figure 1a. A new honeycomb core, called Taiji hexagon cell structure is proposed and used in this research (Figure 1b). This structure has been fortified in the basement of the traditional hexagonal one. Every typical unit has added a Taiji curve inside of the cell and strengthened to three layers of paper between cells boundaries while the traditional hexagonal one only has two layers (Figure 1). The characteristics of the paper raw material used to prepare the Taiji honeycomb core are shown in Table 2 according to ASTM D1037-06a.

The adhesive to attach the surface layers and the correspondent honeycomb core was polyvinyl acetate (PVAc) from Franklin International Company (Columbus, OH, USA). It was also used to join craft paper together to form a honeycomb structure.

### 2.2. Specimen Fabrication

**Figure 2:**
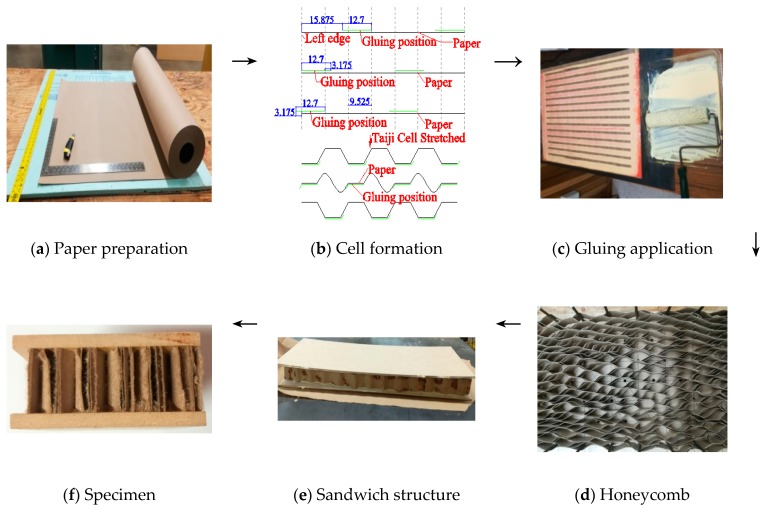
Fabrication of wooden sandwich beam specimens with Taiji honeycomb core.

**Figure 3:**
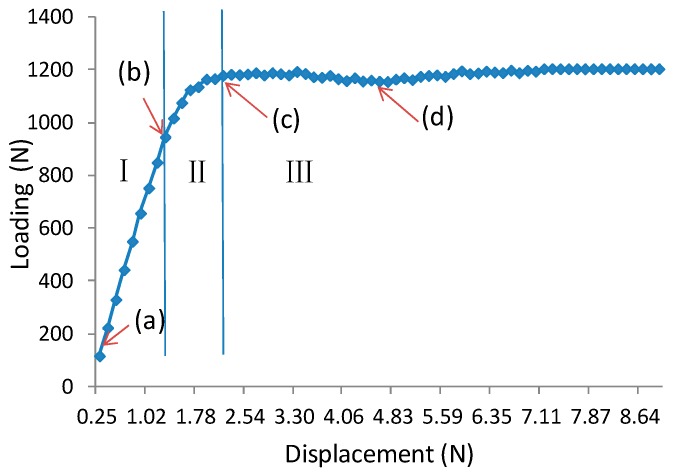
Typical load-displacement curve of core shear failure of wooden sandwich beam with Taiji honeycomb core (A3B2C2): (**a**): Unloaded; (**b**) buckling of inclined cell wall; (**c**) buckling of whole cell wall; (**d**) post-buckling stage.

Kraft paper as presented in Figure 2a, was first cut to a size of 508 × 635 mm. Then 12.7 mm width of gluing strips were positioned periodically as presented in Figure 2b with distances of 28.575 mm (3 × 9.525 mm) along the paper edge. After PVAc glue was applied on the designated position of the papers (Figure 3c), three paper pieces were stacked with sequences with offset distance to the edge of −3.175 mm, 0 and 15.875 mm, respectively, and then repeated until up to 48 layers. After that, the stacked paper was pressed under 0.5 MPa for 4 h. According to specific experiment measurements, stacked papers were sliced vertically to strips of 15.875 mm, 25.4 mm and 34.925 mm. The honeycomb strips were stretched and fixed under temperature 85 °C for 10 min. Then sandwich panel was assembled manually by bonding wooden face sheets (MDF and PLY) to a paper honeycomb core, using room-temperature-curing glue of 240 g/m^2^. After that, a compression load of 0.1 MPa was applied on those sandwich beams and kept for 4 h to form strong internal strength of composite. To minimize the size effect of the honeycomb structure, all specimens were cut to the width of 58 mm (Figure 2d–f).

### 2.3. Test Methods

To identify as many failure modes as possible, specimen combinations of various face sheets and core thickness were tested under a three-point bending. The experiments performed are presented in Table 3, and all of them were carried out using a universal testing machine (MTS systems) in displacement control, setting the crosshead speed according to Equation (1). The diameter of the cross head was 76.2 mm. Span distances were varied in a range between 76.2 mm to 381 mm to assess the independence of the critical loading on it. Recently, digital image correlation method was applied to precisely observe the deformation and failure process of wood-based materials [24,25,26], so this method was also involved in this paper to measure strain distribution close to the loading roller.

(1) $$\;N=\frac{0.005L^{2}}{6h}\;$$

where N is the speeding rate of loading; h is the composite thickness and L is the effective length (span distance) of the sandwich beam.

## 3. Result and Discuss

### 3.1. Failure Process of Sandwich Beam (Experimental Results)

**Figure 4:**
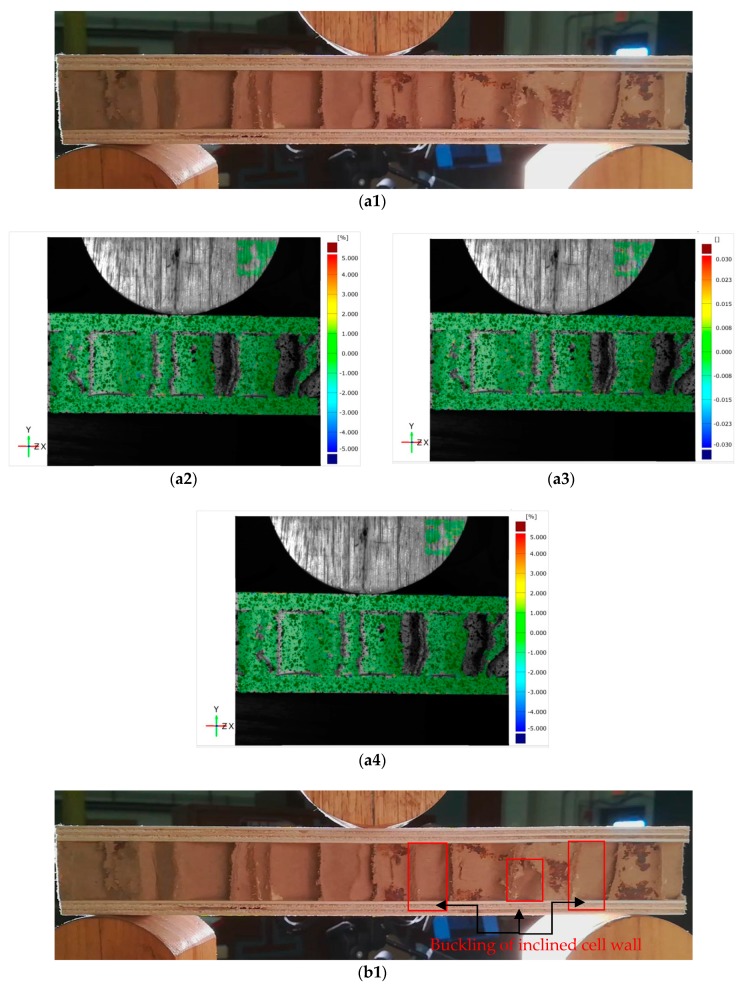

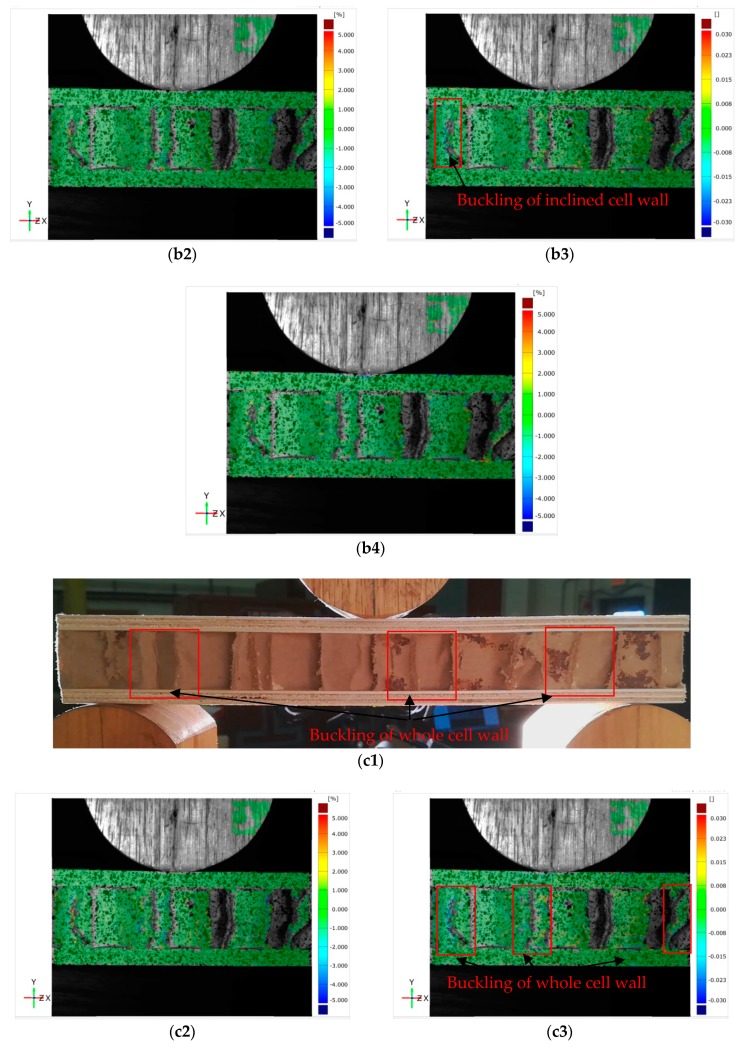

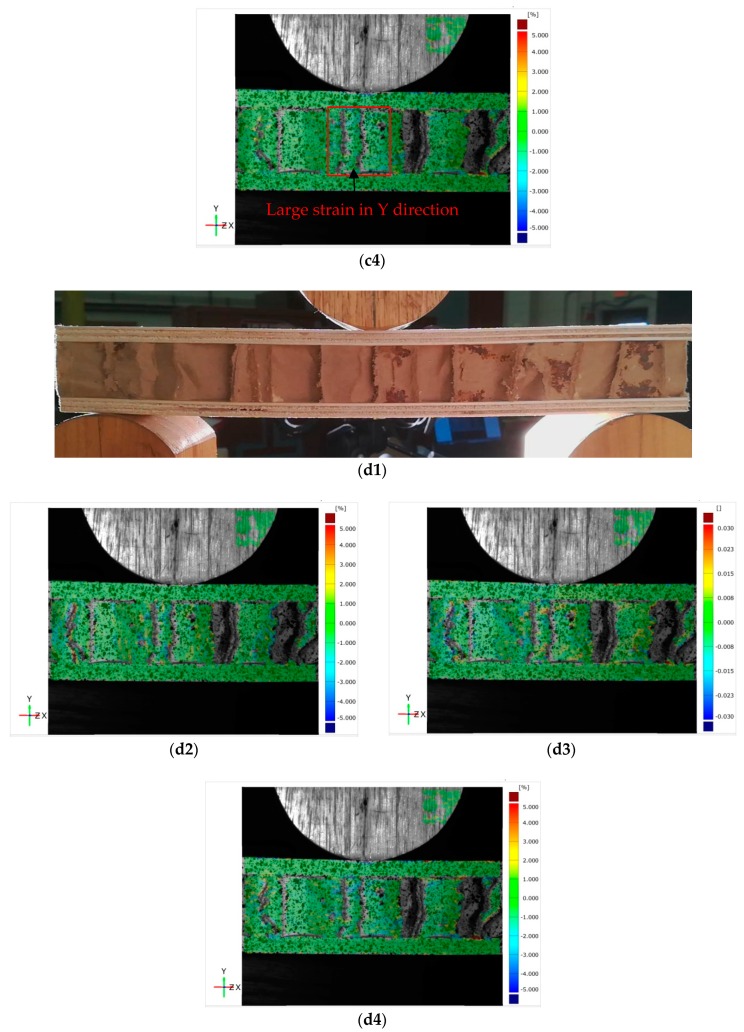
Photographs and strain distribution of core shear failure process of wooden sandwich beam with Taiji honeycomb core: **a1** for photograph, **a2** for $\epsilon_{x}$, **a3** for $\gamma_{xy}$ and **a4** for $\varepsilon_{y}$ under unloaded condition; **b1** for photograph, **b2** for $\epsilon_{x}$, **b3** for $\gamma_{xy}$ and **b4** for $\varepsilon_{y}$ under buckling of inclined cell wall; **c1** for photograph, **c2** for $\epsilon_{x}$, **c3** for $\gamma_{xy}$ and **c4** for $\varepsilon_{y}$ under buckling of whole cell wall; **d1** for photograph, **d2** for $\epsilon_{x}$, **d3** for $\gamma_{xy}$ and **d4** for $\varepsilon_{y}$ under post-buckling stage.

**Figure 5:**
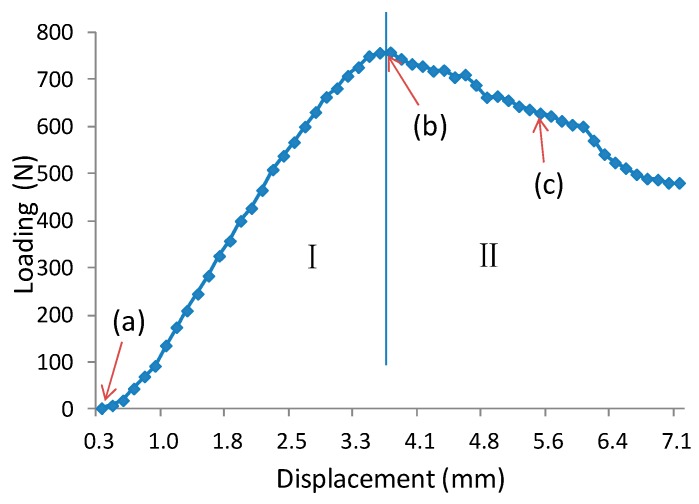
Typical load-displacement curve of indentation process of wooden sandwich beam with Taiji honeycomb core (A1B3C3): (**a**) unloaded; (**b**) initiation of indentation; (**c**) showing condition after indentation.

**Figure 6:**
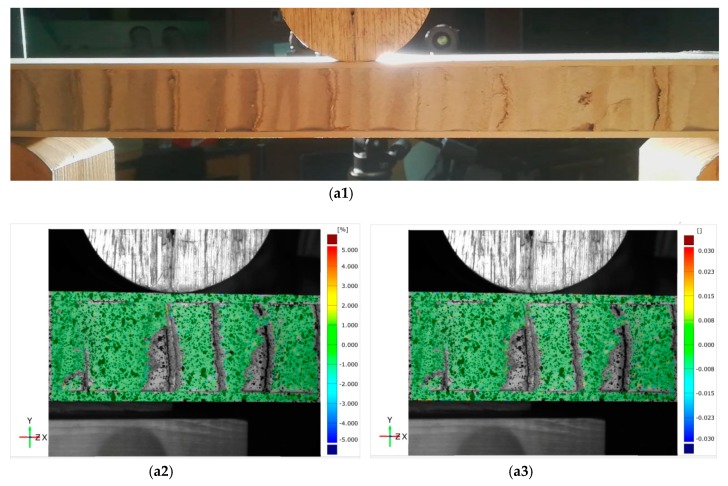

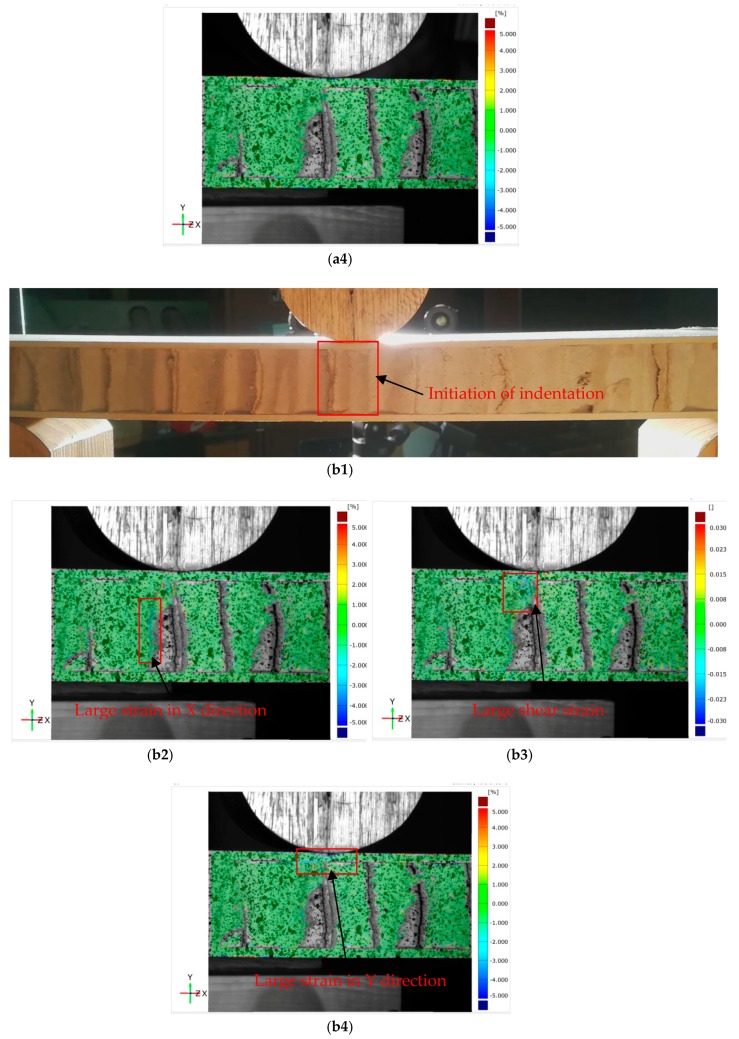

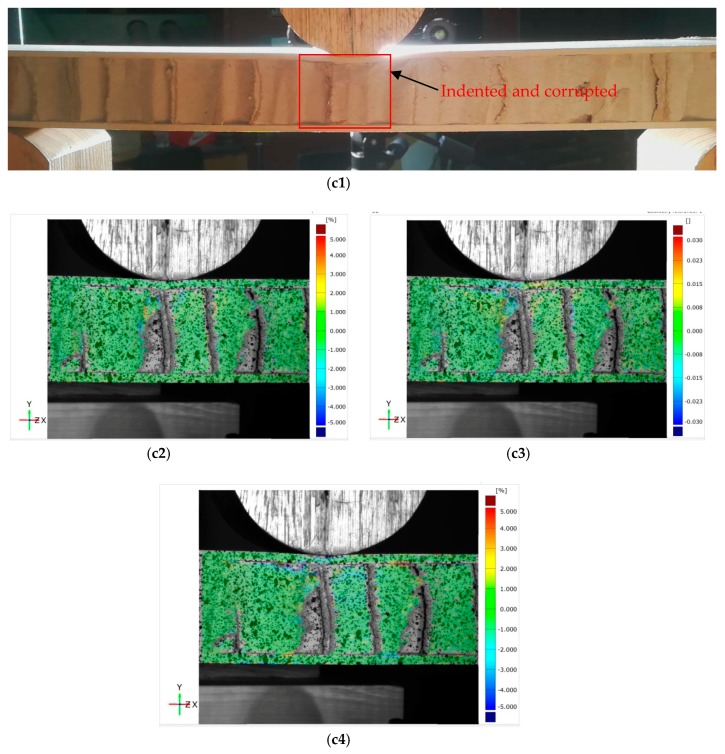
Photographs and strain distribution of the indentation process of the wooden sandwich beam with Taiji honeycomb core: **a1** for photograph, **a2** for $\epsilon_{x}$, **a3** for $\gamma_{xy}$ and **a4** for $\varepsilon_{y}$ under unloaded condition; **b1** for photograph, **b2** for $\epsilon_{x}$, **b3** for $\gamma_{xy}$ and **b4** for $\varepsilon_{y}$ under initiation of indentation; **c1** for photograph, **c2** for $\epsilon_{x}$, **c3** for $\gamma_{xy}$ and **c4** for $\varepsilon_{y}$ after indentation.

In general, sandwich beams under three-point bending test fail according to the following modes: face yielding, face wrinkling, core shear, and indentation [27,28,29]. The failure depends on the properties of each layer, their geometrical dimensions, and the loading conditions. For a wooden sandwich beam with a paper honeycomb core, wrinkling was not generally observed due to the relatively low strength of the core to the faces; typical face yield was also not observed due to the limited thickness-span ratio of specimens. In this experiment, core shear and indentation were the two dominant failure modes. There were two typical deformation patterns in terms of failure modes, which are presented in Figure 3, Figure 4, Figure 5 and Figure 6.

Figure 3 exhibits a typical load-displacement curve of core shear failure for a wooden sandwich beam with a Taiji honeycomb core. The core shear failure can be approximately categorized into three stages, named as I, II, III. In the first stage I, the correlation between loading and transverse displacement is almost linear, which confirms the Hook’s law. The load increases linearly until the shear stress of core has attained the buckling point of the inclined cell wall with large shear strain (Figure 4(b1–b4)), then, the deformation goes into stage II. In this stage, the ribbon cell wall remains carrying an increasing load while the inclined wall only takes the same level of bulking load, or even a portion of buckling load. Thus, the loading elevates nonlinearly to the maximum point (Figure 4(c1–c4)) when the ribbon wall starts buckling; then stage III starts. Large compression strain of Y direction ($\varepsilon_{y}$) underneath the loading roller was also observed along with shear buckling. The loading level lasts for a period of time, which is called plateau loading (Figure 4(d1–d4)). According to [30], there probably had stage IV that the fracture on the interface between core and surface layer will be occurred due to too much shear deformation in the honeycomb core, thus the loading level will decline sharply.

Unlike the core shear failure, typical load-displacement curves of the indentation process of the wooden sandwich beam with Taiji honeycomb core are represented in two stages, named as I and II (Figure 5). In stage I, the elastic deformation of the sandwich beam is observed, and the load rises linearly to peak along with the increasing of the roller-head displacement. It should be noted that the slope of the curve tends to decrease with raised deflection in the end of stage I which reveals the existence of plastic deformation. After the load is beyond the maximum, the indentation initiates, exhibiting a complicated strain distribution that $\varepsilon_{y}$ integrates with $\epsilon_{x}$ and $\gamma_{xy}$ (Figure 6(b1–b4)), and the load begins to decrease step by step, which is called stage II. In this stage, the local bending of the surface under the roller head was observed and corrupted honeycomb core was compressed (Figure 6(c1–c4)). However, the sandwich beam can also carry a load to some extent in this stage unless the local break occurred in the surface sheet. Interestingly, the previous behaviors are in close agreement with Caprino’s research [13].

### 3.2. Failure Load Prediction of Sandwich Beam

**Figure 7:**
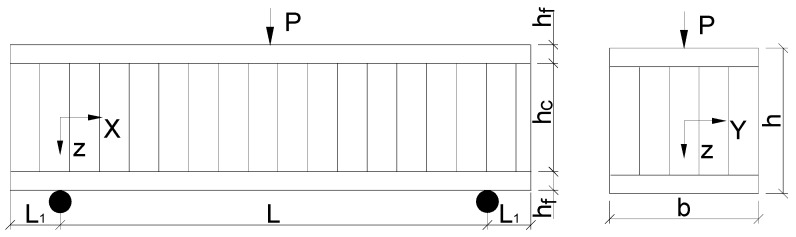
Specimen notation considered for the analytical models.

As mentioned in the literature, the most common failure patterns for sandwich beams are shear failure, indentation and face yield. In this section, analytical models were used to predict the strength of the sandwich beam with Taiji honeycomb core, under a three-point bending test. The specimen considered in the analysis is graphically represented in Figure 7, which consisted of a sandwich beam of span length L and width b. The specimen has two identical face-sheets of thickness $h_{f}$ and a paper honeycomb core of thickness $h_{c}$. The model’s results will be compared with experimental data.

#### 3.2.1. Shear Failure

Core shear failure occurred when the shear stress reached a critical value of core material [27].

(2) $$\;P_{cr}=k_{1}\tau_{cs}2b\left(h_{c}+h_{f}\right)\;$$

with $k_{1}=\left(1+\frac{L_{1}}{L}\right)$

where, $P_{cr}$ is critical loading when core shear failure occurred,$\;\tau_{cs}\;$ is the shear strength of the honeycomb core, $k_{1}$ is the coefficient to adjust the overhang effect on shear strength, $L_{1}$ is the overhang length of the sandwich beam (Figure 7).

#### 3.2.2. Indentation

Indentation is a common local failure mode in bending tests of sandwich composites subject to central point loading. Indentation occurs when the stress under roller attains compression strength of the core. To predict the failure load, it is theoretically assumed that the elastic skin is resting on an ideal elastic foundation that represents the core as a system of independent and linear elastic springs according to Winkle’s hypothesis. The solution was given [31]:

(3) $$\;P_{cr}=\frac{2b\sigma_{cr}}{\lambda }\;$$

with $\lambda^{2}=\sqrt{\frac{E_{cz}}{4E_{f}I_{f}h_{c}}}$

where, $\sigma_{cr}$ denotes the transverse compression strength of the core, $E_{f}I_{f}$ is the bending rigidity of the surface and $E_{cz}$ is the transverse elastic modulus of the core.

However, Equation (3) is not considering the effect of global bending stress on local indentation failure, and thus, the solution overestimates the critical load if the span distance of three-point bending is large. Recently, [28] proposed a model considering both the local central loading and global bending stress:

(4) $$\;P_{cr}=\frac{Lb}{2}\sigma_{cr}\left(-\frac{\sigma_{cr}}{E_{cz}}+\sqrt{\frac{\sigma_{cr}^{2}}{E_{cz}^{2}}+32\sqrt{\frac{E_{f}I_{f}/L^{4}}{E_{cz}/\left(h_{c}+h_{f}\right)}}}\right)\;$$

Except for elastic solution, the plastic model proposed by [32] is also extensively used for prediction of critical loading.

(5) $$\;P_{cr}=bh_{f}\sqrt[{3}]{\frac{\pi \left(h_{f}+h_{c}\right)E_{f}\sigma_{cr}^{2}}{3L}}\;$$

#### 3.2.3. Face Yield

Face yield occurs when compressed face sheet attains crush strength under bending. Neglecting the contribution load associated with deflection of the core, the collapse load of the sandwich beam is then given by [27]:

(6) $$\;P_{cr}=\frac{4bh_{f}\left(h_{f}+h_{c}\right)\sigma_{tr}}{L}\;$$

where, $\sigma_{tr}$ is the tension strength of surface sheets.

### 3.3. Mechanical Prediction of Taiji Honeycomb Core

From Equations (2) to (6), we can observe that transverse compression strength ($\sigma_{cr}$), elastic modulus ($E_{cz}$), and transverse shear strength ($\tau_{cs}$) of honeycomb; in addition to geometry parameters and skin tension strength ($\sigma_{tr}$), are affecting significant to the whole mechanical properties of the sandwich beam. Therefore, the deep understanding of honeycomb characteristics are key points to its entire strength of sandwich structure. Due to the periodical cell construction constituted by the thin wall, the strength of the core is not decided by its strength of material, but the buckling stress of the structure. The cell collapse can be recognized as buckling of the interconnected thin wall with spring constraints between the honeycomb prism under transverse compression or shear.

#### 3.3.1. Compression Buckling Stress

The buckling stress of the thin plate is given by Timoshenko [33] as:

(7) $$\;\sigma_{pc}=\frac{K_{c}E_{s}}{\left(1-v_{s}^{2}\right)}{\left(\frac{t}{l}\right)}^{2}\;$$

where, $\sigma_{pc}$ denotes compression buckling stress of thin plate; $E_{s}$ and $v_{s}$ are transverse modulus and Poisson’s ratio of thin plate, respectively; and t and l are respectively thickness and side length of thin plate. $K_{c}\;$ is an end constraint factor that equals 3.29 for simple support and 5.73 for clamp edge when the ratio of height to side length is bigger than 3.0. As for the honeycomb structure, the constraint between the cell walls is neither completely free nor rigidly clamped, as an approximation, [34] gave the value $K_{c}=4.0$. In this paper, $K_{c}=5.0$ was used, which shows a good prediction with measured results.

In the case of Taiji cells, as depicted in Figure 1a, the equivalent compress buckling stress of the representative cell can be expressed as:

(8) $$\;\sigma_{tc}\left(l+lcos\theta \right)2lsin\theta =4lt\frac{K_{c}E_{s}}{\left(1-v_{s}^{2}\right)}{\left(\frac{t}{l}\right)}^{2}+\frac{1}{2}l2t\frac{K_{c}E_{s}}{\left(1-v_{s}^{2}\right)}{\left(\frac{2t}{l}\right)}^{2}+l3t\frac{K_{c}E_{s}}{\left(1-v_{s}^{2}\right)}{\left(\frac{3t}{l}\right)}^{2}\;$$

Therefore, $\sigma_{tc}$ is given as:

(9) $$\;\sigma_{tc}=\frac{17.5}{\left(1+cos\theta \right)sin\theta }\frac{K_{c}E_{s}}{\left(1-v_{s}^{2}\right)}{\left(\frac{t}{l}\right)}^{3}\;$$

where, $\sigma_{tc}$ denotes the compression buckling stress of Taiji honeycomb.

#### 3.3.2. Shear Buckling Stress

Using the similar stability theory, shear buckling stress for a thin wall can be expressed as:

(10) $$\;\tau_{ps}=\frac{K_{s}E_{s}}{\left(1-v_{s}^{2}\right)}{\left(\frac{t}{l}\right)}^{2}\;$$

where, $\tau_{ps}$ denote shear buckling stress of thin plate, $K_{s}\;$ is the boundary constraint factor in shear mode. For the honeycomb structure, considering the size effect of honeycomb, the approximation of 7.7, 6.5 and 5.0 are used in accordance with core thicknesses of 15.875 mm, 25.4 mm and 34.925 mm respectively in this paper.

As for the Taiji cell, shear buckling stress of a representative cell is:

(11) $$\;\tau_{ts}\left(l+lcos\theta \right)2lsin\theta =4lt\frac{K_{s}E_{s}}{\left(1-v_{s}^{2}\right)}{\left(\frac{t}{l}\right)}^{2}cos\theta +\frac{1}{2}l2t\frac{K_{s}E_{s}}{\left(1-v_{s}^{2}\right)}{\left(\frac{2t}{l}\right)}^{2}cos\theta +l3t\frac{K_{c}E_{s}}{\left(1-v_{s}^{2}\right)}{\left(\frac{3t}{l}\right)}^{2}\;$$

Therefore, $\tau_{ts}$ is given as:

(12) $$\;\tau_{ts}=\frac{\left(13.5+4cos\theta \right)}{\left(1+cos\theta \right)sin\theta }\frac{K_{s}E_{s}}{\left(1-v_{s}^{2}\right)}{\left(\frac{t}{l}\right)}^{3}\;$$

where, $\tau_{ts}$ denotes shear buckling stress of Taiji honeycomb.

#### 3.3.3. Compression Modulus

The equivalent compress modulus of a representative cell is:

(13) $$\;E_{tc}=\frac{4}{\left(1+cos\theta \right)sin\theta }\frac{t}{l}E_{s}\;$$

where, $E_{tc}$ denote compression modulus of Taiji honeycomb.

### 3.4. Analytical Comparison between Taiji Honeycomb and Traditional Hexagonal One

The compression buckling stress of traditional hexagonal honeycomb structure, described in Figure 1b, is:

(14) $$\;\sigma_{dc}=\frac{5}{\left(1+cos\theta \right)sin\theta }\frac{K_{c}E_{s}}{\left(1-v_{s}^{2}\right)}{\left(\frac{t}{l}\right)}^{3}\;$$

Thus, the relative compression strength of Taiji honeycomb to a traditional hexagonal one is given by:

(15) $$\;\frac{\sigma_{tc}}{\sigma_{dc}}=3.5\;$$

The shear buckling stress of a traditional hexagonal honeycomb structure is:

(16) $$\;\tau_{ds}=\frac{\left(4+cos\theta \right)}{\left(1+cos\theta \right)sin\theta }\frac{K_{s}E_{s}}{\left(1-v_{s}^{2}\right)}{\left(\frac{t}{l}\right)}^{3}\;$$

Therefore, the relative shear strength of a traditional hexagonal honeycomb is given by:

(17) $$\;\frac{\tau_{ts}}{\tau_{ds}}=\frac{13.5+4cos\theta }{4+cos\theta }\;$$

As for standard hexagonal, that is $\theta =\frac{2\pi }{3}$, Equation (17) can be simplified as:

(18) $$\;\frac{\tau_{ts}}{\tau_{ds}}\approx 3.44\;$$

Next, we will consider the effect of density on the strength of the honeycomb structure. For the traditional hexagonal honeycomb, the relative density is:

(19) $$\;\rho_{d}^{*}=\frac{2}{\left(1+cos\theta \right)sin\theta }\frac{t}{l}\;$$

where $\rho_{d}^{*}$ is the relative density of the traditional hexagonal honeycomb structure to solid ones.

In the case of Taiji cells, as depicted in Figure 1, the relative density is:

(20) $$\;\rho_{t}^{*}=\frac{4}{\left(1+cos\theta \right)sin\theta }\frac{t}{l}\;$$

where $\rho_{t}^{*}$ is the relative density of the Taiji honeycomb structure to solid ones.

Using Equations (19) and (20), we can get relative compress strength of the two-honeycomb structures:

(21) $$\;\frac{\sigma_{tc}}{\sigma_{dc}}=1.75\;$$

And the relative shear strength of the two-honeycomb structures is:

(22) $$\;\frac{\tau_{tc}}{\tau_{ds}}=\frac{13.5+4cos\theta }{8+2cos\theta }\;$$

As for standard hexagonal, that is $\theta =\frac{2\pi }{3}$, Equation (22) will be simplified as:

(23) $$\;\frac{\tau_{tc}}{\tau_{ds}}\approx 1.72\;$$

### 3.5. Comparison between Experiment and Analytical Solution

The predicted and tested results of sandwich beam with Taiji honeycomb core are summarized in Table 4. The failure was taken to be the maximum load carried by the specimen before abrupt load drop and is coincident with the observation of a clearly evident failure. Two failure modes of core shear buckling and local indentation occurred in this experiment while typical surface yield was not observed due to a much lower strength of the core than that of the surface sheets and limited span distance. Core Shear Failure Solution (CSS, Equation (2)) based on Reissner hypothesis, Elastic Solution of Indentation (ES, Equation (3)) by Gdoutos, Elastic Solution of Indentation Considering Bending Stress (ESBS, Equation (4)) by Hao, and Plastic Solution of Indentation Considering Bending Stress (PSBS, Equation (5)) by Steeves and Fleck was applied to estimate the results. The facesheet characteristics as input parameters, are from Table 1 while $\;\sigma_{tc}$,$\;\tau_{ts}$, $E_{tc}$ is obtained from Equations (9), (12) and (13) respectively. Except for shear prediction which has good agreement with measured failure load, elastic solution of ES and ESBS for indentation prediction underestimates the tested results while plastic solution of PSBS overestimates measured failure load, and therefore, the indentation of sandwich beam with paper Taiji honeycomb core was recognized as one failure behavior between elastic and plastic. Therefore, the adjusted solution of ESBS multiply 1.5 was used to fit the experiment, which appears to be in good agreement with the measured results.

### 3.6. The Parametric Effect on Failure Load

**Figure 8:**
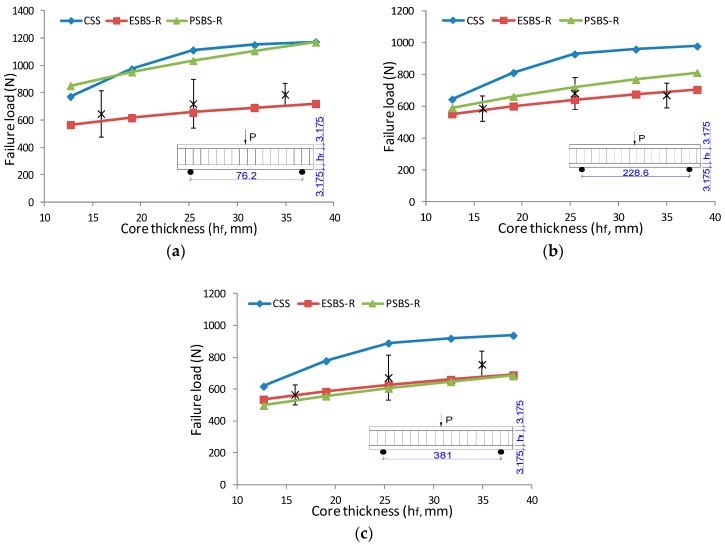
The effect of core thickness on failure load under a three-point bending test: (**a**) Sandwich beam with 3.175 mm MDF face under span of 76.2 mm; (**b**) sandwich beam with 3.175 mm MDF face under span of 228.6; (**c**) sandwich beam with 3.175 MDF face under span of 381 mm.

**Figure 9:**
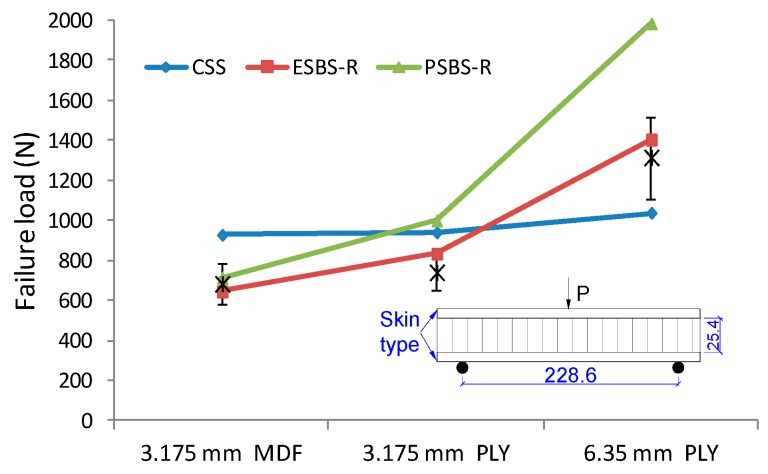
The effect of face sheets type on failure load of sandwich beam under three-point bending.

To understand the effect of construction parameters on the critical value of sandwich beam under three-point bending, the curve of failure load versus core thickness and surface sheets are presented in Figure 8 and Figure 9. The solid line is the failure load predicted by models of CSS, ESBS-R, and PSBS-R (see models at 3.4 above). The asterisk printed in the graph was the tested result for specimens with different geometry combinations. Critical loading increases linearly with core thickness. As core thickness increased from 15.875 mm to 34.925 mm, the maximum loading increased 21.8%, 14.1% and 33.4% for a sandwich beam under 76.2 mm, 228.6 mm and 381 mm span distance, respectively. The surface sheet type also has significant influence on failure load. The critical load of the sandwich beam with 6.35 mm PLY face almost doubled compare to the ones with 3.175 mm MDF face.

**Figure 10:**
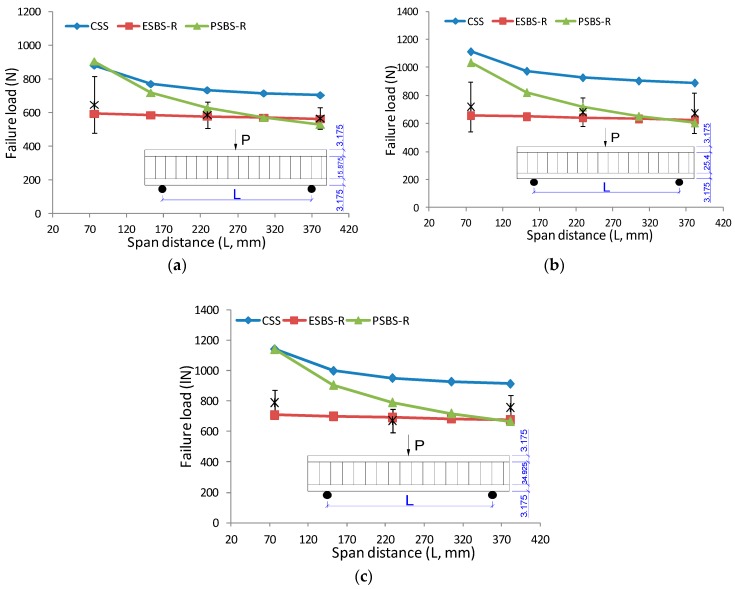
The effect of span distance on failure load under three-point bending: (**a**) Sandwich beam with 3.175 mm MDF face and 15.875 mm core; (**b**) sandwich beam with 3.175 mm MDF face and 25.4 mm core; (**c**) sandwich beam with 3.175 mm MDF face and 34.925 mm core.

Figure 10 exhibits the effect of span distance on the failure load of the sandwich beam under a three-point bending test. The lines represent the model predictions and asterisks are the discrete measured results according to the span distances of 76.2 mm, 228.6 mm and 381 mm. As the span distance increased from 76.2 mm to 381 mm, the maximum loading decreased 12.4%, 6.5% and 4.1% for specimens with 15.875 mm, 25.4 mm and 34.925 mm core thickness, respectively. It should be noted that the reduction is more significant in the sandwich beam with the thin core than the one with the thick core. The reason is that the beam with the thin core has a large global transverse deflection before collapse, and therefore, the bending stress in indented face will give more force in the vertical direction, which accelerates the indentation failure process.

### 3.7. Failure Map of Sandwich Beam with Taiji Honeycomb Core

By combining Equations (3), (4) and (6) with each other to eliminate critical loading ($P_{cr}$), three theoretical curves that delimit the experimental regions of the two types of failure modes are obtained,

(24) $$\;\frac{h_{f}}{L}=\frac{1}{2}\frac{\tau_{cr}}{\sigma_{tr}}\;$$

(25) $$\;\sqrt{\frac{4}{3}}\sqrt{\frac{E_{f}}{E_{cz}}}\frac{h_{f}}{L}\sqrt{\frac{h_{f}}{h_{f}+h_{c}}}=\frac{8}{9}\frac{h_{f}+h_{c}}{L}\frac{\tau_{cr}^{2}}{\sigma_{cr}^{2}}+\frac{2}{3}\frac{\tau_{cr}}{E_{cz}}\;$$

(26) $$\;\sqrt{\frac{h_{f}}{h_{f}+h_{c}}}\sqrt{\;\frac{E_{f}}{E_{cz}}\;}=\frac{8}{9}\frac{h_{f}}{L}\frac{h_{f}+h_{c}}{L}\frac{\sigma_{tr}^{2}}{\sigma_{cr}^{2}}+\frac{1}{3}\frac{\sigma_{tr}}{E_{cz}}\;$$

By defining non-dimensional indexes $\bar{h_{f}}=\frac{h_{f}}{L}$, $\bar{h_{c}+h_{f}}=\frac{\left(h_{c}+h_{f}\right)}{L}$, we can get:

(27) $$\;\bar{h_{f}}=\frac{1}{2}\frac{\tau_{cr}}{\sigma_{tr}}\;$$

(28) $$\;\sqrt{\frac{4}{3}}\sqrt{\frac{E_{f}}{E_{cz}}}\bar{h_{f}}\sqrt{\frac{\bar{h_{f}}}{\bar{h_{f}+h_{c}}}}=\frac{8}{9}\bar{h_{f}+h_{c}}\frac{\tau_{cr}^{2}}{\sigma_{cr}^{2}}+\frac{2}{3}\frac{\tau_{cr}}{E_{cz}}\;$$

(29) $$\;\sqrt{\frac{\bar{h_{f}}}{\bar{h_{f}+h_{c}}}}\sqrt{\;\frac{E_{f}}{E_{cz}}\;}=\frac{8}{9}\bar{h_{f}}\frac{\sigma_{tr}^{2}}{\sigma_{cr}^{2}}\bar{h_{f}+h_{c}}+\frac{1}{3}\frac{\sigma_{tr}}{E_{cz}}\;$$

**Figure 11:**
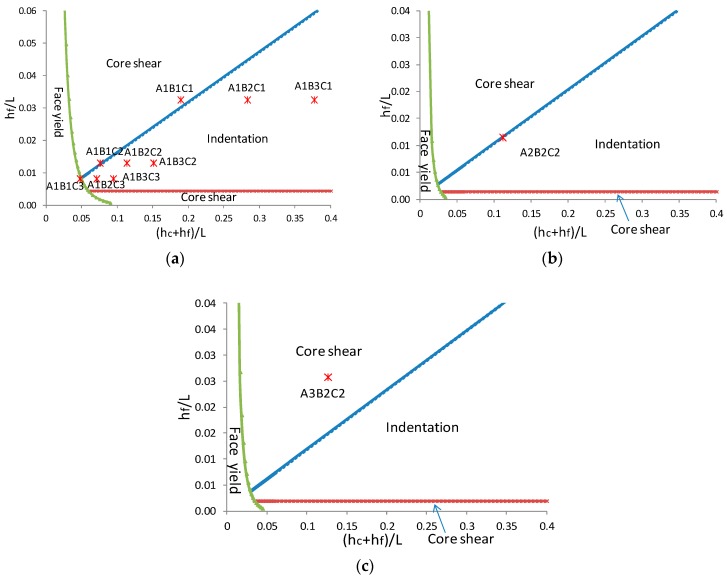
Failure map based on construction parameters: (**a**) sandwich beam with 3.175 mm MDF surface, (**b**) sandwich beam with 3.175 mm PLY surface, (**c**) sandwich beam with 6.35 mm PLY surface.

A transition in failure mode occurs when two mechanisms present the same failure load (Figure 11). The coordinate system is the construction parameters of the sandwich beam, which is (*h_c_* + *h_f_*)/*L* for horizontal axis and *h_f_*/*L* for vertical axis; thus, all possible beam geometries are graphed for a given material combination. In this study, the experiments concentrate on a wooden sandwich composite with a paper honeycomb core and various geometrical parameters. Those material properties have been characterized (Table 1 and Table 2). It should be noted that the core properties are $\sigma_{cr}=\sigma_{tc}=0.22\;\mathrm{MPa}$ (Equation (9)) and $\tau_{cs}=\tau_{ts}=0.25\;\mathrm{MPa}$ (Equation (12)),$\;E_{cz}=E_{tc}=19.5\;\mathrm{MPa}$ (Equation (13)), that are fixed to the average values without considering the side effect or core thickness.

**Figure 12:**
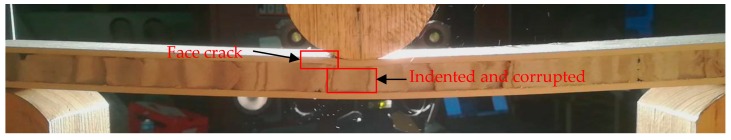
Photograph of indentation combined with face yield under three-point bending.

The predicted failure mode in Figure 11 has good agreement with the observed results summarized in Table 4. The diagram was divided into three fields that were separated by a transition line. The collapse of the sandwich beam is generally decided by one of the competing mechanisms that depends on the geometry of the panel and the mechanical properties of the face and core materials. Under three-point bending test, only if the beam thickness-span ratio is very small, the face yield will have occurred; otherwise indentation and core shear failure will play the dominant role. However, it should be noted that the failure mode of the beam with strong face sheets and thin core thickness tends to core shear buckling (A3B2C2). On the contrary, that with relatively weak face sheets and thick core thickness tends to local indentation (A1B2C1, A1B3C1, A1B2C2, A1B3C2, A1B2C3, A1B3C3). When construction parameters were close to the transition line, it was possible to have two failure modes or three (A1B1C2, A1B1C3, A2B2C2), which demonstrated that those types of composites are at a failure mode transition area. However, sometimes combined failure mode can be observed in the transition area; Figure 12 exhibited indentation and face yield occurred with the specimen code of A1B1C3. There also was an exception. Specimens of A1B1C1 only had indention failure observed in the experiment despite of the closure to the transition line between shear and indentation. The reason is shear failure load elevated significantly due to the enhancement of the overhang effect that was also proved in the research of [35].

Laminate characters also have a significant effect on failure mode. Specimens of A1B2C2 with 3.175 mm MDF surface have significant indentation collapse while A2B2C2 with 3.175 mm PLY moves the boundary between indentation and core shear failure due to the relatively high strength of face sheets but same with other structure parameters.

## 4. Conclusions

A new type of Taiji honeycomb structure proposed in this paper has 3.5 times the compression strength and 3.44 times the shear strength of commercial hexagonal ones by treating the cell wall as an interconnected thin plate to calculate the stability capacity. Considering the density effect, the novel structure also has an increase in compression strength of 1.75 times and shear strength of 1.72 times. The reinforcement of the core also results in an almost linear increase to the whole strength of the sandwich composite.

Both the experiment and theoretical methods were employed to investigate the deformation behavior and the failure mechanism for the sandwich beam with Taiji honeycomb core. The maximum resistance to central loading was improved significantly depending on the increasing skin or core thickness. Surface indentation and core shear failure played the dominant role under three-point bending test conditions. For a large skin thickness-span and beam thickness-span ratio, core shear failure occurred first; otherwise, indentation could occur prior to any core failure. However, typical face yield was not observed due to the limited thickness-span ratio of specimens. Large spans weaken the loading level due to the contribution of global bending stress in the compressive skin to indentation failure. As the span distance increases, the maximum loading declines accordingly regardless of core thickness. Using representative unit analysis method, a set of analytical equations between key structure parameters and properties were developed to accurately predict the threshold stresses corresponding to the onset of those deformation events.

## Figures and Tables

**Table 1:** Properties of the medium density fiber board (MDF) and plywood (PLY) used in the outer layers (skins).

Material	Thickness (mm)	Density (Kg/m^3^)	Moisture Content (%)	Bending Strength (MPa)	Bending Modulus (MPa)
MDF	3.175	869.0	5.4	28.9	5399.9
PLY	3.175	683.6	5.6	88.2	20,578.0
PLY	6.35	672.5	5.4	64.2	13,598.7

**Table 2:** Paper characteristics to fabricate the honeycomb core.

Material	Thickness (mm)	Moisture Content (%)	Tensile Strength (MPa)	Tensile Modulus (MPa)
Kraft paper	0.1778	5.4	13.2	453.0

**Table 3:** Experimental parameter combinations of sandwich beam structures.

Group	Code	Surface Sheet (A)	Core Thickness (B, mm)	Span Distance (D, mm)
1	A1B1C1	3.175 mm MDF	15.875	76.2
2	A1B2C1	3.175 mm MDF	25.4	76.2
3	A1B3C1	3.175 mm MDF	34.925	76.2
4	A1B1C2	3.175 mm MDF	15.875	228.6
5	A1B2C2	3.175 mm MDF	25.4	228.6
6	A1B3C2	3.175 mm MDF	34.925	228.6
7	A2B2C2	3.175 mm PLY	25.4	228.6
8	A3B2C2	6.35 mm PLY	25.4	228.6
9	A1B1C3	3.175 mm MDF	15.875	381
10	A1B2C3	3.175 mm MDF	25.4	381
11	A1B3C3	3.175 mm MDF	34.925	381

**Table 4:** Estimated and measured failure load of sandwich beam under three-point bending.

Group	Code	Surface Sheet (A)	Core Thickness (B, mm)	Span Distance (D, mm)	Fail Mode	Test Results (N)	Standard Deviation	CSS (N)	ES (N)	PSBS (N)	PSBS-R (N)	ESBS (N)	ESBS-R (N)
1	A1B1C1	3.175 mm MDF	15.875	76.2	Indentation	647.9	170.1	﹨	409.4	1125.4	893.1	420.1	629.7
2	A1B2C1	3.175 mm MDF	25.4	76.2	Indentation	721.2	178.3	﹨	541.1	1186.4	941.6	438.3	657.3
3	A1B3C1	3.175 mm MDF	34.925	76.2	Indentation	789.0	83.4	﹨	591.0	1344.8	1067.1	482.8	724.5
4	A1B1C2	3.175 mm MDF	15.875	228.6	Indentation	587.8	77.7	﹨	473.5	762.3	605.2	384.0	576.3
5	A1B2C2	3.175 mm MDF	25.4	228.6	Indentation	682.9	101.8	﹨	522.9	880.2	712.0	429.9	644.8
6	A1B3C2	3.175 mm MDF	34.925	228.6	Indentation	670.7	78.5	﹨	588.3	1007.9	801.0	468.1	702.2
7	A2B2C2	3.175 mm PLY	25.4	228.6	Indentation/Core shear	739.6	90.3	939.8	687.5	1258.0	998.6	555.8	833.5
8	A3B2C2	6.35 mm PLY	25.4	228.6	Core shear	1314.1	206.4	1037.3	﹨	﹨	﹨	﹨	﹨
9	A1B1C3	3.175 mm MDF	15.875	381	Indentation with face yield	567.5	63.0	﹨	482.4	705.8	560.3	392.0	587.8
10	A1B2C3	3.175 mm MDF	25.4	381	Indentation	674.6	141.7	﹨	553.1	815.2	647.0	439.7	659.5
11	A1B3C3	3.175 mm MDF	34.925	381	Indentation	756.9	81.7	﹨	585.6	870.9	691.1	463.2	694.6

CSS: Core shear failure solution based on Reissner hypothesis, ES: Elastic solution by Gdoutos, PSBS: Perfect plastic solution considering bending stress by Steeves and Fleck, PSBS-R: PSBS results divided by $\sqrt[{3}]{2}$, ESBS--Elastic solution considering bending stress, ESBS-R: ESBS results multiply 1.5.
